# Supplementary material for: The footprint of campaign strategies in Farsi Twitter: A case for 2021 Iranian presidential election
Source: PLoS One. 2022 Jul 28;17(7):e0270822. doi: 10.1371/journal.pone.0270822 (PMC9333222; doi:10.1371/journal.pone.0270822)
Supplement: S1 Appendix — (PDF) [file pone.0270822.s001.pdf]

**S1 Appendix. Trending hashtags.** In table 2, the trending hashtags about the election during the data gathering process is listed with the specific label given to them, which identifies the context of the hashtag. If a hashtag is used to advertise for a candidate (My vote Jalili), it is labelled with the name of the candidate(Jalili). If it is used to discredit a candidate (Raisi the killer of 67), it is labelled with an anti and the name of the candidate (Anti-Raisi).

| **No.** | **Translation of the hashtag** | **Label** |
|---|---|---|
| 1 | My vote is Jalili | Jalili |
| 2 | Saeed Jalili | Jalili |
| 3 | Jalili | Jalili |
| 4 | Ghalibaf | Ghalibaf |
| 5 | Haj Bagher | Ghalibaf |
| 6 | The chosen best | Ghalibaf |
| 7 | Ali Motahari | Motahari |
| 8 | Mohsen Rezaii | Rezaii |
| 9 | Raisi | Raisi |
| 10 | Saeed Mohammad | Mohammad |
| 11 | Vote no Vote | Anti |
| 12 | We are waiting for the election | Pro |
| 13 | No to the Islamic Republic | Anti |
| 14 | The big unity | Pro |
| 15 | The revolutionary economist | Rezaii |
| 16 | We are all one | Pro |
| 17 | No to the repeat of Rouhani | Pro |
| 18 | The two people | Pro |
| 19 | Raisi or Ghalibaf | Pro |
| 20 | The queue of change | Pro |
| 21 | My vote | Pro |
| 22 | The enthusiasm of choice | Pro |
| 23 | The horror in history making | Pro |
| 24 | The triangle of change | Pro |
| 25 | The vigilant guides | Pro |
| 26 | The candidate with a plan | Rezaii |
| 27 | The young revolutionary government | Mohammad |
| 28 | What are you guarding | Anti |
| 29 | The garden of attendance | Pro |
| 30 | Thank you Raisi | Raisi |
| 31 | The central vote | Pro |
| 32 | Rostam Ghasemi | Ghasemi |
| 33 | Action and change | Ghalibaf |
| 34 | Raisi the killer of 67 | Anti Raisi |
| 35 | Either Ahmadi or nobody | Ahmadinejad |
| 36 | Larijani | Larijani |
| 37 | Iran's hope | Mohammad |
| 38 | Ahmadinejad | Ahmadinejad |
| 39 | My vote is Rezaii | Rezaii |
| 40 | Unity for improvement | Pro |
| 41 | The unity of races | Pro |

| | | |
|---|---|---|
| 42 | The excitements of Khuzestanis | Pro |
| 43 | The unity of the revolutionary front | Pro |
| 44 | The land of keeper of the Velayat | Pro |
| 45 | Mr. Mohsen | Rezaii |
| 46 | We are waiting for the election | Pro |
| 47 | I will vote | Pro |
| 48 | Barandazam (The movement to dismantle the Regime) | Anti |
| 49 | Hemmati | Hemmati |
| 50 | The government of Jihad and Leap | Jalili |
| 51 | Boycott Iran Sham Election | Anti |
| 52 | My vote is destruction | Anti |
| 53 | Raisi is the voice of people | Raisi |
| 54 | Zakani | Zakani |
| 55 | Debate | Debate |
| 56 | The debate of 1400 | Debate |
| 57 | Raisi of the Judiciary tell us about the trial of the Corruptors of the stock | Pro |
| 58 | Mehralizadeh | Mehralizadeh |
| 59 | Ghazizadeh Hashemi | Ghazizadeh |
| 60 | Ghazizadeh is the voice of the soldier | Ghazizadeh |
| 61 | Abdolnasser Hemmati | Hemmati |
| 62 | The anti corruption government | Raisi |
| 63 | Rezaii the winner of debates | Rezaii |
| 64 | The request for the leader's order | Ahmadinejad |
| 65 | The field of choice | Pro |
| 66 | Fake candidate | Anti Hemmati |
| 67 | Policing for Managers | Raisi |
| 68 | The resistance of Bueen Zahra | Pro |
| 69 | Iranians Boycott Elections | Anti |
| 70 | Salam | Ghazizadeh |
| 71 | The People's government The strong Iran | Raisi |
| 72 | The bipartisan justice | Raisi |
| 73 | The powerfull government | Raisi |
| 74 | The cosmical rudness | Anti Hemmati |
| 75 | The people of the field | Pro |

| | | |
|---|---|---|
| 76 | The king of bank bribary | Anti Hemmati |
| 77 | Just five vote | Pro |
| 78 | No 2 IR | Anti |
| 79 | The public Consensus candidate | Pro |
| 80 | The spoken union | Pro |
| 81 | The public's insight | Pro |
| 82 | For the love of Imam Reza | Pro |
| 83 | I voted | Pro |
| 84 | Creating Embarrassment | Anti |
| 85 | Duty again | Pro |
| 86 | The public's dignity | Pro |
| 87 | The party of Iran's public | Pro |
| 88 | I did not vote | Anti |
| 89 | The nationwide strikes | Anti |

**Table 2.** The list of the translation of the trending hashtags about the election during the data gathering process. The labels given to each hashtag is based on the contexts of the tweets that used it. It should be noted that 97 hashtags were monitored. Since some of them were only different in either language or one letter, only the 89 unique hashtags are listed here.
