# Supplementary material for: The footprint of campaign strategies in Farsi Twitter: A case for 2021 Iranian presidential election
Source: PLoS One. 2022 Jul 28;17(7):e0270822. doi: 10.1371/journal.pone.0270822 (PMC9333222; doi:10.1371/journal.pone.0270822)
Supplement: Attachment — Submitted filename: Response to Reviewers.pdf [file pone.0270822.s003.pdf]

Response to the reviewers

We thank the reviewers for their critical assessment of our work as their constructive criticism has improved the work immensely. In the following we address their concerns point by point. The authors detailed answers are written in blue, and the pieces added to the article are written in color red.

Reviewer 1

Reviewer Point P 1.1 — The manuscript argues that it intends to analyze organized activities on Persian Twitter, but it did not do so as the data and analyses did not provide sufficient and substantial evidence of such activities. It is the major shortcoming of the current work. In fact, findings are quite descriptive. The findings section mainly provides a descriptive account of the rise and fall of communities in Persian Twitter.

- Moreover, it indicates that most of these communities were populated by bots. While these findings are interesting, authors need to go deeper to investigate more substantial questions and shed light on more complicated dynamics of bots' activism on Persian Twitter. Moreover, it is not enough to simply state that Twitter communities were home of bots and automated accounts. You need to give more details, e.g., how much of nodes in each cluster were bot? which factors affect the number of bots in each community? What is the ratio of bots to genuine users in each cluster? Then you should discuss what these numbers mean in terms of organized activities. Authors also mentioned time to time that some changes in the network, such as disappearing a community or merging communities with each other, are the evidence of organized activities on Persian Twitter (for instance; lines 211-213). However, they did not provide enough information to support their arguments. It is not enough to just mention that there are some coordinated actions based on the number of bots or the changes of communities. You should go deeper to explore how these bots worked and were constituted.

  Reply: We agree with the reviewer that knowing the cap score of all the nodes within a cluster would provide us with a clearer depiction of bot activity within them, however, the Rapid API that is used to collect cap score is highly limited (only 500 requests per day). To solve this issue, The cap score of the top 10 percent of most retweeted users that fortunately account for more than 90% of the edges within the networks was gathered to demonstrate what type of users were responsible for the majority of activities in the discussion. We attempted to gather the cap score of all users in later days and discovered that most of the accounts have either gotten suspended or deleted, which would not be an accurate representation of the time of data collection and reinforces the notion of non-genuine activity within all clusters. Furthermore, in all of the communities except for Hemmati's, there is no genuine user among the top retweeted users, thus the ratio of bots to genuine users would not provide any information.

  The following paragraph is added to the discussion in lines 279-288:

  Considering the evidence of the high level of bot activity in all communities alongside other observations such as the way some communities were working together to gain support and leave the discussion in favour of the other at a sensitive point, all point to an organised campaign from

all parties involved, to attempt to control the public opinion. In a non-political event, this number of bots and such actions could lead us to an advertising campaign that wants to gain its audience's attention. However, this level of bot activity during an important political event, like an election, could only point to organisations or political groups coordinating actions to manipulate public opinion. Our results suggest that multiple political groups were organising such activities in this case.

- The manuscript should provide more details on how organized activities on Persian Twitter were operated and to which extent? For instance, how and by what candidates/parties were bots employed? To which extent? Were there any similarities/differences between bots activism in different communities? How did automated accounts interact with other users to manipulate the flow of content? How did they try to intervene with genuine users' practices in shaping counter narratives and discourses?

  Reply: Our results suggest that bot activism is very similar in all communities. The average cap score of top retweeted users in all communities is similarly about 0.8 which considering these users are responsible for more than 90% of retweets, suggests a considerably high bot activity in all the parties involved. One noticeable difference is in the fake follower's score. It is apparent in figure 7 that Hemmati's community has a lower score than others, which could point to the higher rate of genuine users within this community.

  The following paragraph is added to the discussion in lines 273-278:

  As it is mentioned before, a small number of nodes have more than 90%of the edges, creating what can be regarded as a core that is responsible for the network structure and the flow of information to a cloud that has a more significant number of users with much less activity where it can be assumed most of the genuine users exists. Further analysis suggests that "cores" in the opposite poles do not interact. However, they do interact within a pole in different communities.

- The conclusion section raises several points which were not supported by data and analyses.

  - In the first paragraph, you suddenly mentioned the Covid-19 pandemic. Since it is not the focus of your research, and you did not discuss it before, I suggest consider removing it.

    Reply: The Covid-19 pandemic was mentioned to signify the importance of social media in this election where no physical events were held and instead the resources of the campaigns were focused on social media. However, the authors agree with the reviewer that this statement in the conclusion is not necessary and removed it.

  - In this section, you made some points about the anonymity of users and raised some abrupt arguments about the anonymity of users on Persian Twitter. First, I can't see how it is relevant to your research. Furthermore, the problem of anonymity of users on Persian Twitter is more complicated than can be concluded in a few lines. I suggest to base your conclusions on your data and analyses. None of these conclusions were supported by your work.

    Reply: This section was removed

– The manuscript mentioned in lines 296-298: our current study of the debate network of the Iran 2021 election reveals that the main competition was not between the presidential candidates but between two large isolated poles, one encouraging people to vote and the other for voter suppression. I am wondering why authors did not consider that this could be pertinent to the fact that most of candidates were deprived from participating in election by the Guardian Council. In fact, the competition was not between candidates since most of them were belonged to a same political camp which was supported by the state power.

Reply: The lines 314-323 are edited as follows:

Unlike other elections, our studies reveal that the competition was not between the presidential candidates but between two large isolated poles, one encouraging people to vote and the other for voter suppression. Most of the candidates worked together in favor of the president-elect. This indicates that the opposition party did not have an acceptable candidate in the election, and most of the candidates in the race were from the right-wing (who were strongly supported by the state). This is a notable difference in this election compared to the previous Iranian election. By merely studying the retweet network of the election, one could recognize the structure of the competition within the election is telling of how the Persian Twittersphere is representative of the political system in Iran.

– You argued in line 285-287: Our study of the Iran 2021 election indicates that the Farsi Retweet network is an effective instrument to identify political debate, analyze and even predict political dynamics and campaign strategies. Your research did not carry out these jobs. For instance, how did your work analyze campaign strategies?

Reply: The following campaign strategies can be derived from the analysis.

* There is a concentrated population of bots within each community that accounts for more than 90 percent of the retweets, which points to a coordinated movement to control and manipulate the propagation of information from all parties involved.
* A large number of these accounts are deleted after the election, this number is much higher in the Pro community where most of the candidates and the president–elect's community exist. It appears that the strategy of the pro community was to participate and attempt to control this particular discussion and did not intend to continue to participate after the discussion ended, which is also apparent from the sudden fall in the number of users in the Pro community, a week after the election.
* The appearance of the Anti-Hemmati community; It is demonstrated that during the debates, the priority of the Pro community was to propagate information in opposition to Hemmati. During which a considerable number of users, did not show support for their candidate and only posted anti Hemmati hashtags.
* A significant number of users, migrate from Jalili's community to Raisi's. This occurs two days before Jalili ends his campaign. A similar movement is observed in Mohammad's community prior to that. This is indicative of the fact, that the goal of Mohammad's and Jalili's campaign was to gain support for Raisi.

Reviewer Point P 1.2 — Authors should provide more context on politics and Twitter in Iran. They discussed several candidates' communities in findings section, but these candidates

are unknown to the readers who are not familiar with Iran's political landscape. Furthermore, they should discuss the political atmosphere in which 2021 election was held. For instance, the disapproval of many reformist candidates which made many citizens angry. Or the nationwide protests in November 2019 which affected the rate of participation and the discussions on social media significantly.

Reply: A significant difference in the Iranian election from other countries is the presence of the Anti community. The lack of a viable representative for the opposition and censorship, have made Twitter a platform to voice the opposition's opinion. As Khazraee (2019) and Kermani et al (2021) pointed out Twitter has been used as an outlet for freedom of speech for many Iranians. The social activism on Twitter has increased significantly after certain events such as the green movement and as the reviewer has correctly mentioned the protest in 2019. This resulted in noticeable political engagement on Farsi Twitter and simultaneously the rise of organized activities to control and manipulate the public.

The following paragraph is added to the introduction in the lines 41-50.

It is important to mention, the 2021 Iranian presidential election was between the following candidates from the conservative party; Ebrahim Raisi (president-elect), Saeed Jalili (withdrew in favor of Raisi), Alireza Zakani (withdrew in favor of Raisi), Mohsen Rezaii and Amir Hossein Ghazizadeh, and the following candidates from the reformist party; Abdolnaser Hemmati and Mohsen Mehralizadeh (withdrew in favor of Hemmati). It is important to note that the candidacy of many politicians was rejected most of whom were from the reformist party which resulted in certain groups boycotting the election. The following politicians were rejected a presidential candidacy and were outspoken during the election. Mahmud Ahmadinejad and Saeed Jalili.

Reviewer Point P 1.3 — I can't agree more with authors in this fact that there is an academic gap in analyzing coordinated activities on Persian Twitter. But I believe there are many studies in this topic on other countries. Authors should review the existing literature and link this work to them. In this way, they can show what is the added value of the current study. Doing so, they will also able to highlight the contribution of this research to this field beyond Persian Twitter.

Reply: The following paragraph is added to the introduction in lines 11-22.

Coordinated activities by automated agents to propagate a political agenda on Twitter were first detected during the 2010 Massachusetts election [10]. Other notable examples are; the Russia-related influence operation to manipulate voters in the US 2016 presidential election [11], the influence of social bots in the Brexit referendum [12], and the alt-right campaign to influence the 2017 French presidential election [13]. These organized acts have been the subject of many studies, with the intent to detect such activities and reveal the more vulnerable communities [14, 15]. Studies usually resort to comprehensive statistical and content analysis while still being unable to achieve their goal in many cases [16–20]. While network analysis has been used to detect online manipulation [21–24] and to detect the active communities within twittersphere [25], in this article, we were able to go one step further and detect organizational activities and identify the communities involved with such activities, through network analysis.

Reviewer Point P 1.4 — Authors need to compare and contrast the findings with those analyzing previous presidential elections, such as Kermani and Adham (2021) work on 2017 election, and Khazraee (2019) work on 2013 election. They should discuss how the community of users has been transformed during the time to highlight the novelty of their findings.

Reply: Both these studies are mentioned in the introduction and one important novel discovery is the rise of pro-government users within the Persian Twittersphere which was not true in the previous works and is indicative of the evolution of Farsi Twitter. These works focused mainly on the genuine users' activities and eliminated bot activities, while this article focuses on online manipulation, hence, in each community, there is a high presence of automated agents. Therefore, the communities in previous works are not compared to the communities in this article, as the users within these communities are fundamentally different.

Reviewer Point P 1.5 — Authors provide comprehensive details about the methodology. However, I suggest to remove some parts of this section if they could be found in reference books. If they devised a new approach or measurement, then it should be clearly mentioned. Instead, I suggest to discuss what these measurements mean and what they did in this research.

Reply: The following paragraph is added to the method in lines 116-124 and the suggested parts are removed.

Some of the network's essential structural characteristics are measured to study and compare the structure of different communities. These properties include; Average clustering coefficient[39], which gives us an insight into the linking structure within a community; modularity[37], which measures the presence of smaller clusters within a community; the average shortest path, that would indicate the small worldness of a community and assortativity[40], measures each node's likelihood to link with similar nodes, for instance, the probability of a hub linking to another hub node. These properties would provide a vivid representation of the structural differences and similarities of different communities.

Reviewer Point P 1.6 — I also suggest to get involved more critically with literature into social media activism and coordinated action to disrupt it.

Reply: The following paragraph is added to the introduction in lines 3-9:

Initially, social media was seen as a way to amplify political and cultural grievances throughout the world, its role in organizing protests and giving a voice to the opposition in instances like the Arab Spring [1], the Green movement [2], the "Me Too" movement [3] and the Black Lives Matter movement [4], portrayed social media as a "liberation technology" [5]. The transparency provided by social media created a sense of trust in users to use these platforms for political means [6].

Reviewer Point P 1.7 — I guess the columns of No. users, and No. tweets should be exchanged in Table 1. They seem wrong now.

Reply: The table is corrected.

Reviewer Point P 1.8 — MEK is a militia group. Authors need to provide more information on this group, and also mention what this abbreviation stands for.

Reply: The following sentence was added to the discussion in lines 175-176.

Mojahedin E Khalq or People's Mojahedin, is a militia group located outside of Iran that advocates overthrowing the Iranian regime

Reviewer Point P 1.9 — Several sentences need proper references throughout the paper.

Reply: We appreciate the Reviewer's precision. Some references were added in lines 127, 128, and 155.

Reviewer Point P 1.10 — I don't understand this sentence: Note that this number differs from node's in-degree since the network is constructed from only the tweets that include a trending hashtag about the election (85-86).

Reply: The data collection is done by searching a specific hashtag and collecting every tweet containing that hashtag at a specific time. Node's in-degree only represents the number of times a specific users' tweets got retweeted during that specific time, which differs from the retweet number, that is the collective number of retweets for a specific user overall.

The sentence is edited as follows:

Note that this number differs from the node's in-degree since the network is only representative of the specific time period and the retweet count of the user's tweets represents the number of retweets overall.

Reviewer 2

Reviewer Point P 2.1 — The introduction should be at least three paragraph: motivation, the paper's contribution in the larger literature, and the data and results of the paper. Much of this information is in the one introductory paragraph and just needs to be split.

Reply: The authors appreciate the precision of the reviewer and edited the introduction. The first paragraph is a literature review of the organized activity on social media platforms. The second paragraph is a literature review of the previous works about Farsi Twitter. In the fourth paragraph, the goal and the method of the study are discussed. A short summary of the results is given in the fifth paragraph.

Reviewer Point P 2.2 — I have questions about the data collection. First, I would like to see a table, in the appendix is fine, of the 97 hashtags. Second, I need to know more. Is it Twitter that identified if they are trending? Is 97 all of the trending hashtags during the period? Third, did the authors connect to the streaming API, query a search endpoint in real time, or use the Academic Research product after the fact? Fourth, why divide the period into weeks instead of aggregating by days? There are enough users and tweets to analyze the data by day instead of by week.

Reply: We used a third-party website that spans the Twitter data and identify the trending Hashtags on Farsi Twitter (Sabamedia [is out of service now], and trendsmap.com). Afterward, the hashtags regarding the election are labeled and gathered. The streaming API is used to collect the data by querying a specific hashtag and then every tweet containing that word is collected in an SQLite database, subsequently, the users' data are gathered in a separate SQLite database and the PostgreSQL, which works as a relational database, is updated. Initially, we started by creating the networks of single days, as the reviewer mentioned, hoping to get an accurate picture of the changes over time. Shortly after, we realized users mostly engage about the election after a specific event, like the debates or the process of candidate validation. We concluded that a network of a single day would not provide much information as the events instigating a conversation on Twitter would not occur in a day-to-day frequency, The periods in which, the data for the network is accumulated are shorter in the week of the election.

However, a shorter time span for the weeks prior to the election would not provide much information in comparison to a week-by-week analysis.

The following table is added to the appendix.

No.	Translation of the hashtag	Label
1	My vote is Jalili	Jalili
2	Saeed Jalili	Jalili
3	Jalili	Jalili
4	Ghalibaf	Ghalibaf
5	Haj Bagher	Ghalibaf
6	The chosen best	Ghalibaf
7	Ali Motahari	Motahari
8	Mohsen Rezaii	Rezaii
9	Raisi	Raisi
10	Saeed Mohammad	Mohammad
11	Vote no Vote	Anti
12	We are waiting for the election	Pro
13	No to the Islamic Republic	Anti
14	The big unity	Pro
15	The revolutionary economist	Rezaii
16	We are all one	Pro
17	No to the repeat of Rouhani	Pro
18	The two people	Pro
19	Raisi or Ghalibaf	Pro
20	The queue of change	Pro
21	My vote	Pro
22	The enthusiasm of choice	Pro
23	The horror in history making	Pro
24	The triangle of change	Pro
25	The vigilant guides	Pro
26	The candidate with a plan	Rezaii
27	The young revolutionary government	Mohammad
28	What are you guarding	Anti
29	The garden of attendance	Pro
30	Thank you Raisi	Raisi
31	The central vote	Pro
32	Rostam Ghasemi	Ghasemi
33	Action and change	Ghalibaf
34	Raisi the killer of 67	Anti Raisi
35	Either Ahmadi or nobody	Ahmadinejad
36	Larijani	Larijani
37	Iran's hope	Mohammad
38	Ahmadinejad	Ahmadinejad
39	My vote is Rezaii	Rezaii
40	Unity for improvement	Pro

41	The unity of races	Pro
42	The excitements of Khuzestanis	Pro
43	The unity of the revolutionary front	Pro
44	The land of keeper of the Velayat	Pro
45	Mr. Mohsen	Rezaii
46	We are waiting for the election	Pro
47	I will vote	Pro
48	Barandazam (The movement to dismantle the Regime)	Anti
49	Hemmati	Hemmati
50	The government of Jihad and Leap	Jalili
51	Boycott Iran Sham Election	Anti
52	My vote is destruction	Anti
53	Raisi is the voice of people	Raisi
54	Zakani	Zakani
55	Debate	Debate
56	The debate of 1400	Debate
57	Raisi of the Judiciary tell us about the trial of the Corruptors of the stock	Pro
58	Mehralizadeh	Mehralizadeh
59	Ghazizadeh Hashemi	Ghazizadeh
60	Ghazizadeh is the voice of the soldier	Ghazizadeh
61	Abdolnasser Hemmati	Hemmati
62	The anti corruption government	Raisi
63	Rezaii the winner of debates	Rezaii
64	The request for the leader's order	Ahmadinejad
65	The field of choice	Pro
66	Fake candidate	Anti Hemmati
67	Policing for Managers	Raisi
68	The resistance of Bueen Zahra	Pro
69	Iranians Boycott Elections	Anti
70	Salam	Ghazizadeh
71	The People's government The strong Iran	Raisi
72	The bipartisan justice	Raisi
73	The powerfull government	Raisi
74	The cosmical rudness	Anti Hemmati
75	The people of the field	Pro
76	The king of bank bribary	Anti Hemmati
77	Just five vote	Pro
78	No 2 IR	Anti
79	The public Consensus candidate	Pro
80	The spoken union	Pro
81	The public's insight	Pro
82	For the love of Imam Reza	Pro
83	I voted	Pro
84	Creating Embarrassment	Anti
85	Duty again	Pro

86	The public's dignity	Pro
87	The party of Iran's public	Pro
88	I did not vote	Anti
89	The nationwide strikes	Anti

Table 1: The list of the translation of the trending hashtags about the election during the data gathering process. The labels given to each hashtag is based on the contexts of the tweets that used it. It should be noted that 97 hashtags were monitored. Since some of them were only different in either language or one letter, only the 89 unique hashtags are listed here.

Reviewer Point P 2.3 — The authors claim retweets are discussions. I disagree. A quote retweet could be a discussion, but a retweet just means engagement. The authors should be clearer on this point. Moreover, if they want actual discussion, they could use the conversation ID that Twitter now gives, in v2 of the API, to every tweet and its replies.

Reply: The authors agree with the reviewer, in fact in this article retweet network was used as the structure of the flow of information between users. It is vital for the purpose of the study to analyze the way information regarding the election is propagated between different users, therefore retweets containing hashtags related to the election were gathered through the Twitter API endpoint. The language is corrected according to what the reviewer mentioned.

Reviewer Point P 2.4 — Next, tell me something about the location of who is tweeting; the authors only do so for MEK and then only obliquely. Though Twitter is banned in Iran, there is evidence that many people there use it; see this blog post by Layla Hashemi and Steven Wilson that does a good job of identifying users in Iran:https://www.washingtonpost.com/politics/2020/01/08/twitter-is-where-iranian-dissidents-might-support-soleimanis-killing-opposite-happened/. Two other ideas for location: VPNs do not change users' location, so some of the tweets may actually show up as being from Iran (this is what Roberts, Hobbs, and Steinert-Threlkeld do for their work on China), and look at users' self-reported profile location. You could also look at the timezone of the user profile. At the end of the day, the results are not wrong or right if they are driven by individuals outside of Iran, but knowing that factoid will make the results clearer.

Reply: We were able to collect users' self-reported locations. This analysis was done on the Anti and Pro community separately, to compare the differences within the two poles. To get a relatively accurate result, the terms used in the locations are tokenized and cleaned by stopwords and punctuation. Then the list tags is sorted by the number of times a word has appeared and then we manually extracted the ones referring to an actual real location (Many users have put places like "Hogwarts" or "Hell" as their location). Afterwards, we counted the locations in Iran and out of Iran. The results is as follow:

- In the Anti community which consists of 39341 users. 22120 user have reported a meaningful location, and out of those 6126 users have declared to live in Iran and 3759 have declared to live elsewhere.

- In the Pro community which consists of 77292 users. 45704 user have reported a meaningful location, and out of those 22062 users have declared to live in Iran and 1831 have declared to live elsewhere.

The following is added to the discussion in lines 240-146:

In order to get a better sense of the users behind the tweets, The self-reported locations of the users in each pole is investigated. Figure 1 (c) and (d) demonstrate the portion of users who declared to live in Iran and the ones who declared to live elsewhere in Anti and Pro community respectively. The unidentified group refer to users who either did not report their location, or did not use an actual place for their location (for instance some reported places like, "Hell" or "Hogwarts"). It is evident that users in the Pro community were more likely to declare Iran as their current residence.

Reviewer Point P 2.5 — My biggest critique is that the figures are so blurry they are essentially unreadable.

Reply: Figures 4 ,5 ,6 , and 7 have been corrected and replaced.

Reviewer Point P 2.6 — Finally, there are very many typos that I fear reflect a lack of attention to detail, not English as a second language.

Reply: The authors appreciate the reviewer's feedback and have corrected the issues mentioned in addition to reviewing and editing the whole manuscript.

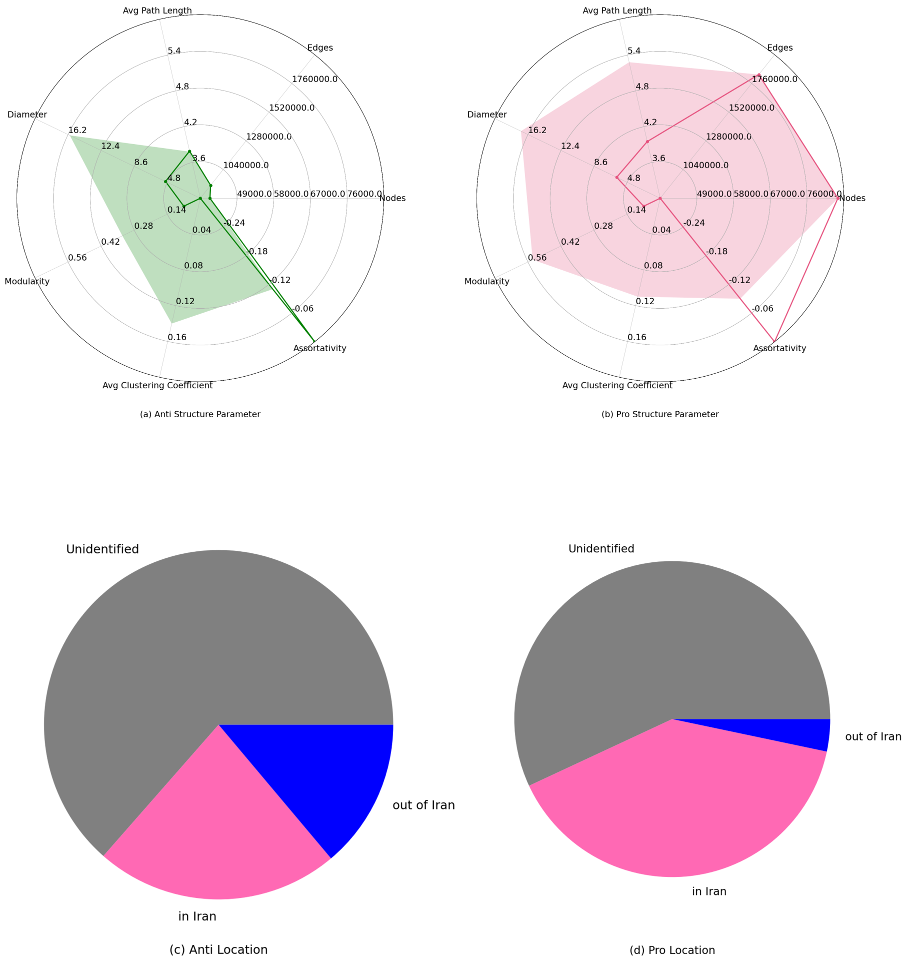

Figure 1: (above) radar graph of structural properties the two main subgraphs in the retweet network. The properties are; number of nodes, number of edges, diameter, average path length, modularity, assortativity, average clustering coefficient. The solid line represents the properties of a random graph with the same amount of nodes and edges. (a) Anti subgraph (b) Pro subgraph. pie graph of the proportion of the users in each community that reported to either live in Iran or elsewhere. (c) Anti subgraph (d) Pro subgraph
