# Supplementary material for: The footprint of campaign strategies in Farsi Twitter: A case for 2021 Iranian presidential election
Source: PLoS One. 2022 Jul 28;17(7):e0270822. doi: 10.1371/journal.pone.0270822 (PMC9333222; doi:10.1371/journal.pone.0270822)
Supplement: Attachment — Submitted filename: Response to Reviewers.pdf [file pone.0270822.s004.pdf]

Response to the reviewers

We thank the reviewer for his critical assessment of our work as his constructive criticism has improved the work immensely. In the following we address his concerns point by point. The authors detailed answers are written in blue, and the pieces added to the article are written in color red.

Reviewer 1

Reviewer Point P 1.1 — First, as I mentioned in the previous round, the focus of this work is currently on the RT network in Persian Twitter during the 2021 election, not the coordinated activities. The findings provide a descriptive account of the RT network and fail to go deeper to investigate the coordinated activities on Persian Twitter. Since the latter is the aim of this work, the focus should be on examining the bot and coordinated actions with more details.

- The authors mentioned in their letter that they found many deleted or suspended accounts. These findings are of significant importance in analyzing coordinated actions. They could detail these findings, discussing how many of the accounts in each community are bots or automated, as well as the portion of the accounts that had been suspended or deleted. Of course, I do not mean they do that on the whole network. They could focus on a particular portion in each cluster. Also, the authors mentioned that the number of bots in all communities is the same. In this step, I suggest they should examine the automated accounts' communicative practices and strategies. They can compare these mechanisms within pro-regime clusters and between pro and anti-regime communities. Such analyses add to the dept of the research and enable authors to provide more substantial statements and conclusions.

  Reply: The following statements is added to the discussion in lines 270-281:

  The users were again examined one week after the data gathering process (2 weeks after the election). It was discovered that 7.53% of the users in the Pro community and 7.16% of the users in the Anti community were either deleted or suspended. The deactivation of this many accounts so close to the trend suggests that such accounts only appeared to participate in the election discussion by either tweeting or retweeting a relevant hashtag which points to online manipulation. Moreover, the average cap score of the 1% most retweeted accounts in the pro and anti-communities are 0.81 and 0.78, respectively. This indicates that in both communities, hub accounts were more likely bots. It should be noted that there are no genuine users (accounts with a cap score of lower than 0.3) in the hubs of the pro community, which shows that the more influential accounts in this community all have automated or manipulatory behavior.

- Also, we know already that MEK and monarchist groups use bots on a large scale on Twitter. Researchers could try to examine if there are other groups using bots in anti-community or not? In each case, they can extend their analyses by comparing the results with the bot activism in the pro-regime cluster.

  Reply: The authors agree with the reviewer that a better examination of the structures behind the organized activities requires a more in-depth look at the subgroups in each cluster. Thus, we

tried identifying known political groups on each pole, i.e., MEK and Pahlavi supporters in Anti and supporters of different political figures in Pro. The data gathering is done through hashtags and different groups in the anti-community used the same hashtags (with exception of Raisi killer of 67, which was mainly used by MEK supporters). Therefore, all users within this cluster are believed to be promoting or expressing the same point (boycotting the election). A more in-depth examination requires a lot more text analysis which can be done manually or through NLP modules, both of which require a lot of resources. The goal of the Anti-community is to instigate a movement for boycotting the election, and the purpose of the Pro community seems to be a campaign for the president-elect. Any sub-organizations within these communities share the same goal and work towards the same result. Therefore, for the purpose of this study, they are unified.

- As another suggestion, the authors mentioned that there are no reciprocal ties between poles. There are mainly in-group relations. Elaborate more on such findings instead of discussing the changes in communities.

  Reply: The following statements is added to the discussion in lines 234-242

  The lack of reciprocal ties between poles reinforces the idea that this is a highly polarized event, in addition to the presence of echo chambers in each pole. There are no interactions between the users of each pole, and most users in this network prefer to interact with like-minded people. This reduces the probability of a user changing their views on the matter. It can be suggested that most of the users in this network, who have either tweeted or retweeted a hashtag relevant to the election, have participated in this discussion aware of their beliefs and with no intention of being influenced by other ideas. This is consistent behavior with users who join the discussion with the agenda to influence others.

Reviewer Point P 1.2 — Moreover, while they mentioned some works on Persian Twitter and coordinated activities in other countries, they did not compare and contrast their result with the existing literature. They should go further than simply mentioning some previous studies. They should clearly show the links between their work with the existing body of research to highlight the contribution of this study.

Reply: The following paragraph is edited in to the introduction in the lines 9-45.

Later on, many studies discovered traces of online manipulation in socio-political events. Researchers suggested that political actors have invested in and used social media platforms to propagate their agenda and manipulate the public in their favor [7–9]. Bessi et al. [10] discovered such activities in the 2016 U.S. presidential election by identifying over 400000 bot accounts on Twitter that participated in the election discussion. Similarly, Bastos et al. [11] focused on the 13493 bot-like Twitter accounts that tweeted during the UK European Union membership referendum debate to identify online manipulation during Brexit.

Other studies have also focused on identifying automated accounts to discover coordinated actions during socio-political events [12–14]. However, bot detection requires sophisticated machine learning algorithms, and comprehensive statistical and text analysis while still in some cases have not been successful [15–19]. Network science has been used along with other statistical approaches to identify the spread of fake news [20], detect voter suppression [21], and echo chambers [22]. In this article, we show that by merely observing the evolution of communities in the retweet network, it is possible to not

only detect some campaign's tactics but also get an overall accurate representation of the prominent political players who competed to gain voter's attention.

The political landscape of Farsi Twitter during Iranian elections in 2013 and 2017 has been investigated by Khazraee [23] and Kermani et al. [24] Both these studies have focused on the genuine users' activities and how voters expressed themselves on Twitter during this time. The increase in the online activities of the conservative party is observed in the 2017 election, however, in both cases, the majority of users are in favor of the reformist party. However, the significant presence and participation of conservative supporters are observed during this election. The majority of users who were involved in the 2021 election discussion have been identified as conservative supporters. These studies gave an overall picture of how Farsi Twitter users interacted and expressed themselves during elections, we attempt to add to these researches by examining the actions of political groups and detecting the presence of online manipulation within this discussion. The presence of political campaigns during elections on Twitter has been heavily studied [25–27]. Election campaigns use different methods like employing fake users, trolls, bots, and sophisticated digital marketing techniques for their operations during elections [10, 12]. Detection of these organized activities and inauthentic users is significant in protecting the integrity of democracies. Farzam et al. [28] used network analysis to identify online manipulation in different types of discussions on Farsi Twitter. There is a lack of research when it comes to organized activity and user manipulation through Farsi Twitter in a consequential era like a presidential election.
