# Supplementary material for: The footprint of campaign strategies in Farsi Twitter: A case for 2021 Iranian presidential election
Source: PLoS One. 2022 Jul 28;17(7):e0270822. doi: 10.1371/journal.pone.0270822 (PMC9333222; doi:10.1371/journal.pone.0270822)
Supplement: Attachment — Submitted filename: PLoS ONE review.docx [file pone.0270822.s002.docx]

Comments on: The Footprint of Campaign Strategies in Farsi Twitter: A case for 2021 Iranian presidential election

This paper aims to investigate coordinated and organized activities in Persian Twitter during the 2021 presidential election. The manuscript is well structured and written. Also, I agree with authors that we need more work on Iranian Twittersphere to better understand how and to what extent organized activities are shaped and operated in this space. Though, I believe that this work has certain potentials to contribute to our knowledge of Twitter activism, by bots and by genuine users, there are some major and minor revisions that should be addressed first. I outline the most important of these revisions below.

- The manuscript argues that it intends to analyze organized activities on Persian Twitter, but it did not do so as the data and analyses did not provide sufficient and substantial evidence of such activities. It is the major shortcoming of the current work. In fact, findings are quite descriptive. The findings section mainly provides a descriptive account of the rise and fall of communities in Persian Twitter. Moreover, it indicates that most of these communities were populated by bots. While these findings are interesting, authors need to go deeper to investigate more substantial questions and shed light on more complicated dynamics of bots’ activism on Persian Twitter. Moreover, it is not enough to simply state that Twitter communities were home of bots and automated accounts. You need to give more details, e.g., how much of nodes in each cluster were bot? which factors affect the number of bots in each community? What is the ratio of bots to genuine users in each cluster? Then you should discuss what these numbers mean in terms of organized activities.

Authors also mentioned time to time that some changes in the network, such as disappearing a community or merging communities with each other, are the evidence of organized activities on Persian Twitter (for instance; lines 211-213). However, they did not provide enough information to support their arguments. It is not enough to just mention that there are some coordinated actions based on the number of bots or the changes of communities. You should go deeper to explore how these bots worked and were constituted. The manuscript should provide more details on how organized activities on Persian Twitter were operated and to which extent? For instance, how and by what candidates/parties were bots employed? To which extent? Were there any similarities/differences between bots activism in different communities? How did automated accounts interact with other users to manipulate the flow of content? How did they try to intervene with genuine users’ practices in shaping counter narratives and discourses? Instead of dealing with such questions that can enhance our understanding of organized activities on Persian Twitter, the manuscript just gave a descriptive explanation of the changes in communities on Twitter.

A related point; since the findings are descriptive, authors were unable to provide some solid and explanative conclusions. The conclusion section raises several points which were not supported by data and analyses. I provide some examples below:

- In the first paragraph, you suddenly mentioned the Covid-19 pandemic. Since it is not the focus of your research, and you did not discuss it before, I suggest consider removing it.
- In this section, you made some points about the anonymity of users and raised some abrupt arguments about the anonymity of users on Persian Twitter. First, I can’t see how it is relevant to your research. Furthermore, the problem of anonymity of users on Persian Twitter is more complicated than can be concluded in a few lines. I suggest to base your conclusions on your data and analyses. None of these conclusions were supported by your work.
- The manuscript mentioned in lines 296-298: our current study of the debate network of the Iran 2021 election reveals that the main competition was not between the presidential candidates but between two large isolated poles, one encouraging people to vote and the other for voter suppression. I am wondering why authors did not consider that this could be pertinent to the fact that most of candidates were deprived from participating in election by the Guardian Council. In fact, the competition was not between candidates since most of them were belonged to a same political camp which was supported by the state power.
- You argued in line 285-287: Our study of the Iran 2021 election indicates that the Farsi Retweet network is an effective instrument to identify political debate, analyze and even predict political dynamics and campaign strategies. Your research did not carry out these jobs. For instance, how did your work analyze campaign strategies?

Other conclusions also should be supported by your data.

Other points:

- Authors should provide more context on politics and Twitter in Iran. They discussed several candidates’ communities in findings section, but these candidates are unknown to the readers who are not familiar with Iran’s political landscape. Furthermore, they should discuss the political atmosphere in which 2021 election was held. For instance, the disapproval of many reformist candidates which made many citizens angry. Or the nationwide protests in November 2019 which affected the rate of participation and the discussions on social media significantly.
- I can’t agree more with authors in this fact that there is an academic gap in analyzing coordinated activities on Persian Twitter. But I believe there are many studies in this topic on other countries. Authors should review the existing literature and link this work to them. In this way, they can show what is the added value of the current study. Doing so, they will also able to highlight the contribution of this research to this field beyond Persian Twitter.
- Authors need to compare and contrast the findings with those analyzing previous presidential elections, such as Kermani and Adham (2021) work on 2017 election, and Khazraee (2019) work on 2013 election. They should discuss how the community of users has been transformed during the time to highlight the novelty of their findings.
- Authors provide comprehensive details about the methodology. However, I suggest to remove some parts of this section if they could be found in reference books. If they devised a new approach or measurement, then it should be clearly mentioned. Instead, I suggest to discuss what these measurements mean and what they did in this research.
- I also suggest to get involved more critically with literature into social media activism and coordinated action to disrupt it.

Some minor points:

- I guess the columns of No. users, and No. tweets should be exchanged in Table 1. They seem wrong now.
- MEK is a militia group. Authors need to provide more information on this group, and also mention what this abbreviation stands for.
- Several sentences need proper references throughout the paper.
- I don’t understand this sentence: Note that this number differs from node’s in-degree since the network is constructed from only the tweets that include a trending hashtag about the election (85-86).

This manuscript has some promises and I do like to see it is out. I wish the authors success in revising the paper to turn it to a more solid work. I think the paper could contribute to Twitter studies in Iran and fill some existing gaps. I know how valuable doing these researches in Iran are, and I do appreciate the authors endeavor in conducting such an innovative and thoughtful study in Iranian context. Finally, I hope the authors find the comments helpful and wish them luck in their academic career.

Best wishes.
